# Neural Correlates and Molecular Mechanisms of Memory and Learning

**DOI:** 10.3390/ijms25052724

**Published:** 2024-02-27

**Authors:** Simone Battaglia, Alessio Avenanti, László Vécsei, Masaru Tanaka

**Affiliations:** 1 Center for Studies and Research in Cognitive Neuroscience, Department of Psychology “Renzo Canestrari”, Cesena Campus, Alma Mater Studiorum Università di Bologna, 47521 Cesena, Italy; alessio.avenanti@unibo.it; 2 Department of Psychology, University of Turin, 10124 Turin, Italy; 3 Neuropsicology and Cognitive Neuroscience Research Center (CINPSI Neurocog), Universidad Católica del Maule, Talca 3460000, Chile; 4 Department of Neurology, Albert Szent-Györgyi Medical School, University of Szeged, Semmelweis u. 6, H-6725 Szeged, Hungary; vecsei.laszlo@med.u-szeged.hu; 5 HUN-REN-SZTE Neuroscience Research Group, Hungarian Research Network, University of Szeged (HUN-REN-SZTE), Danube Neuroscience Research Laboratory, Tisza Lajos krt. 113, H-6725 Szeged, Hungary

## 1. Introduction

Memory and learning are essential cognitive processes that enable us to obtain, retain, and recall information. These factors are crucial for survival, adaptation, and creativity. However, the neural and molecular mechanisms that underlie these cognitive functions are not fully elucidated. For decades, researchers have been fascinated by the neurobiological and molecular basis of acquiring, storing, and retrieving information [1]. Recent neuroimaging technologies have provided valuable insights into underlying neuroanatomical brain circuits [2,3,4,5,6,7]. The amygdala, hippocampus, and prefrontal cortex (PFC) are pivotal for shaping memory and facilitating learning. The amygdala, recognized for its significance in emotional processing, interacts with downstream structures such as the hypothalamus and brainstem regions, influencing the expression of emotionally charged responses [8,9,10]. The inhibitory mechanisms within the amygdala, including specific divisions and nuclei, contribute to memory modulation. The hippocampus, which is essential for spatial navigation and contextual memory, forms direct projections with the infralimbic cortex in the PFC and the basolateral amygdala [11,12]. Distinct subregions of the hippocampus have been implicated in various human behavioral features, highlighting their multifaceted roles in cognitive processes.

The PFC has emerged as a critical hub in the neural circuitry of memory and learning. The dorsomedial PFC supports the long-term storage and retrieval of old memories, whereas the ventromedial PFC forms reciprocal connections with the amygdala and other subcortical structures. This subregion is crucial for modulating responses to stimuli and serves as a relay station for information from limbic and subcortical structures. The anterior and posterior subregions of the ventromedial PFC contribute differently to cognitive processes [13,14,15]. Past research has also underscored the role of the PFC in memory consolidation and retrieval. In particular, the ventromedial PFC plays a vital role in recalling memories during subsequent testing, whereas the dorsolateral PFC is implicated in attentional shifts and short-term memory processes [16,17,18]. This comprehensive understanding of the neural and molecular aspects within these regions enhances our insight into the complex mechanisms underlying memory formation and learning processes. The study of the biological basis of memory and learning requires clear identification of the molecular and cellular changes associated with brain plasticity, as memory formation depends on changes in synaptic efficacy that strengthen the associations between neurons [19]. At the cellular level, we understand that the storage of long-term memory involves gene expression, de novo protein synthesis, and the formation of new synaptic connections.

This Special Issue, “Neural Correlates and Molecular Mechanisms of Memory and Learning” aims to provide a better understanding of various aspects of memory and learning, including the role of neurotransmitters and neuromodulators, the significance of synaptic plasticity, and the possibility of pharmacological interventions to modulate cognitive functions in different contexts. The six papers in this Special Issue offer valuable insights into the complex and diverse nature of cognitive processes. They explore various aspects of memory and learning, such as the role of neurotransmitters and neuromodulators, the significance of synaptic plasticity, and the possibility of pharmacological interventions to modulate cognition. These studies cover a variety of topics, from the effects of multisensory stimulation on memory impairment in mice, to the neuropharmacological modulation of N-methyl-D-aspartate (NMDA), noradrenaline, and endocannabinoid receptors in fear extinction learning. The papers also use a variety of approaches, including animal models, computational models, and clinical studies, to investigate memory and learning processes.

In this Editorial, we will provide a brief overview of the main findings and contributions of each article in this Special Issue, as well as identify knowledge gaps and areas for future research. We hope that this Special Issue will inspire further exploration of the neural correlates and molecular mechanisms of memory and learning, as well as encourage interdisciplinary collaboration among researchers in this fascinating area of neuroscience. Memory and learning are complex and dynamic processes involving multiple brain regions, circuits, molecules, and mechanisms. Understanding how these processes work and how they can be modulated is essential for advancing our knowledge of the brain and its functions, as well as for developing novel strategies for enhancing cognitive performance and treating cognitive disorders. The articles in this Special Issue offer valuable insights into some of the current challenges and advances in this field using different approaches and methods. They also highlight the need for more studies on the role of other neurotransmitters and neuromodulators, the importance of other forms of synaptic plasticity, and the long-term effects of pharmacological interventions on cognitive functions. We hope that readers will find these articles informative, stimulating, and useful for their own research endeavors.

## 2. Special Issue Articles

### 2.1. Memory and Learning in Animal Models

Three articles used animal models to investigate the effects of different interventions on memory and learning [20,21,22]. These studies explored the roles of multisensory stimulation, glucocorticoid receptor antagonism, and recognition memory in modulating hippocampal neurogenesis, synaptic plasticity, and fear-related behaviors. Ravache et al. investigated the effects of multisensory stimulation on memory impairment in a mouse model of obesity [20]. The authors showed that multisensory stimulation reverses memory deficits induced by the lack of adrenergic beta-3 receptor, which is involved in thermogenesis and energy expenditure. They also demonstrated that multisensory stimulation enhanced hippocampal neurogenesis and synaptic plasticity, suggesting that it is a potential mechanism for memory improvement.

Lin et al. examined the effects of RU486, a glucocorticoid receptor antagonist, on traumatic stress-induced fear-related abnormalities in rats [21]. The authors showed that RU486 prevented the development of glucocorticoid dysregulation, anxiety-like behavior, and impaired fear extinction when administered shortly after exposure to traumatic stress. However, RU486 failed to reverse these abnormalities when administered later, indicating the presence of a critical intervention window. Sánchez-Rodríguez et al. explored the synaptic changes induced by recognition memory in the hippocampus [22]. The authors used in vivo electrophysiology to measure excitatory and inhibitory synaptic transmissions in the CA3-CA1 pathway during object recognition memory tasks. They found that recognition memory induces a natural long-term potentiation (LTP)-like increase in both excitatory and inhibitory synaptic transmission, which is dependent on NMDA receptors and endocannabinoid signaling.

### 2.2. Memory and Learning in Computational Models

One study used a computational model to simulate dopamine dynamics and Hebbian mechanisms during probabilistic reversal learning in the striatal circuits. This study demonstrates how the model can reproduce experimental data and account for individual differences in learning performance. Schirru et al. presented a computational model of how the brain learns from rewards and switches between different actions in uncertain situations [23]. The model simulates the activity of dopamine neurons and synaptic changes in striatal circuits during probabilistic reversal learning tasks. The model can reproduce the behavioral and neural data from previous experiments with rats and humans, and it can also explain the individual differences in learning performance based on different dopamine levels and learning rates. This model provides a biologically plausible framework for understanding the neural and molecular mechanisms underlying reward-based learning and decision-making.

### 2.3. Memory and Learning in Clinical Contexts

Two articles addressed neuropharmacological modulation and adverse drug reactions in memory and learning in clinical contexts [24,25]. These papers reviewed the current evidence on how NMDA, noradrenaline, and endocannabinoid receptors regulate fear extinction learning, and they reported a case of fluphenazine-induced neurotoxicity with acute parkinsonism and permanent memory loss. Battaglia et al. reviewed the neuropharmacological modulation of NMDA, noradrenaline, and endocannabinoid receptors in fear extinction [24]. The authors summarized the current evidence on how these receptors regulate synaptic transmission and plasticity in the amygdala and prefrontal cortex during fear extinction. They also discussed the potential therapeutic implications of manipulating these receptors for the treatment of fear-related disorders. De Masi et al. reported a case of fluphenazine-induced neurotoxicity with acute parkinsonism and permanent memory loss [25]. The authors described the clinical features, diagnosis, treatment, and outcome of a patient who developed severe neurological complications after receiving fluphenazine, an antipsychotic drug. They also highlighted the importance of pharmacovigilance and pharmacogenetics in preventing adverse drug reactions.

## 3. Discussion

Studies have investigated various aspects of memory and learning, including the role of neurotransmitters and neuromodulators, the importance of synaptic plasticity, and the potential of pharmacological interventions to modulate cognitive functions. The following are some common themes and findings from previous studies that have shown that various neurotransmitters and neuromodulators, such as dopamine, NMDA, noradrenaline, endocannabinoids, and glucocorticoids, are involved in regulating cognitive processes in different brain regions and circuits. For example, Schirru et al. demonstrated how phasic dopamine changes modulate probabilistic reversal learning in striatal circuits, while Battaglia et al. reviewed how NMDA, noradrenaline, and endocannabinoid receptors modulate fear extinction learning in the amygdala and prefrontal cortex [23,24]. Sánchez-Rodríguez et al. investigated the role of natural LTP-like hippocampal synaptic excitation and inhibition in recognition memory, whereas Battaglia et al. investigated the neuropharmacological modulation of NMDA [22,24], noradrenaline, and endocannabinoid receptors in fear extinction learning. Glutamate is a particularly important neurotransmitter in memory and learning processes, as it is the major excitatory transmitter in the brain and is involved in almost all aspects of cognitive function. Glutamate and glutamate receptors are involved in long-term memory formation as well as LTP, a process believed to underlie memory and learning.

Studies have highlighted how synaptic plasticity, such as LTP and long-term depression, is a key mechanism in memory formation and consolidation. For instance, Sánchez-Rodríguez et al. showed that recognition memory induces natural LTP-like changes in both excitatory and inhibitory synaptic transmission in the hippocampus, while Lin et al. showed that RU486 prevents traumatic stress-induced impairments in synaptic plasticity and fear extinction [21,22]. Synaptic plasticity is a key mechanism that underlies memory formation and learning. The unique plasticity of excitatory glutamatergic synapses is essential for memory formation. Synaptic plasticity mechanisms, such as Hebbian and LTP, are the subject of a number of published studies. For example, Schirru et al. investigated phasic dopamine changes and Hebbian mechanisms in striatal circuits during probabilistic reversal learning [23]. These studies suggest that pharmacological interventions can have beneficial or detrimental effects on memory and learning depending on the timing, dosage, and target of the drugs. For example, Ravache et al. showed that multisensory stimulation can reverse memory impairment in a mouse model of obesity by enhancing hippocampal neurogenesis and synaptic plasticity. In contrast, De Masi et al. reported a case of fluphenazine-induced neurotoxicity with acute parkinsonism and permanent memory loss [25].

Many published papers have investigated the potential of pharmacological interventions to modulate cognitive processes. For example, Lin et al. investigated the effects of RU486 in the treatment of traumatic stress-induced glucocorticoid dysregulation and fear-related abnormalities, whereas Battaglia et al. investigated the neuropharmacological modulation of NMDA, noradrenaline, and endocannabinoid receptors in fear extinction learning [21,24]. The potential for pharmacological interventions to modulate memory and learning processes highlights the importance of understanding the underlying neural correlates and molecular mechanisms of cognitive functions as well as the potential for developing new treatments for memory-related disorders.

These investigations contributed significantly to the understanding of the neural correlates and molecular mechanisms of normal cognitive processes, as well as anxiety disorders. Building on the evolving perspective that anxiety disorders stem from strong associative aversive learning, recent studies proposed innovative therapeutic approaches [26]. These approaches involve a range of drugs that act through diverse neurophysiological mechanisms and potentially alter aversive learning in a long-lasting manner [27,28].

This shift aligns with broader discourse on the complexity and multifaceted nature of memory and learning processes [29,30,31]. These findings suggest that comprehending the neural correlates and molecular mechanisms underlying anxiety disorders, particularly in the context of fear acquisition and extinction, opens new avenues for therapeutic interventions. Moreover, the combination of neuropharmacological adjuvants, such as NMDA agonists and cannabinoids, with noninvasive brain stimulation techniques, such as transcranial magnetic stimulation and transcranial direct current stimulation, offers a promising approach to enhance the effectiveness of existing treatments [32,33,34,35,36,37]. Overall, these articles emphasize the complexity and multifaceted nature of memory and learning processes as well as the significance of understanding the neural correlates and molecular mechanisms underlying these phenomena.

These articles highlight knowledge gaps and future research areas, such as the need for more research on the role of other neurotransmitters and neuromodulators in memory and learning, as well as the need for more research on the long-term effects of pharmacological interventions on cognitive functions. While these six papers provide insights into different facets of memory and learning, questions remain that necessitate additional investigation. These are subjects covered in these six papers that should be investigated further. This Special Issue’s papers have explored the function of neurotransmitters and neuromodulators in the processes of cognitive functions, but many other neurotransmitters and neuromodulators remain to be thoroughly explored in this regard. For example, serotonin and somatostatin have been linked to dysfunctional memory and neurodegenerative diseases, respectively; however, their roles remain unknown [38,39,40,41,42,43,44]. To gain a more comprehensive understanding of the underlying mechanisms, future research should examine the role of other neurotransmitters and neuromodulators in cognition.

This Special Issue comprises some papers that explore the possibility that pharmacological interventions could influence the processes of memory and learning. Nevertheless, further investigation of the enduring impact of these interventions on the cognitive domain is warranted. For instance, Lin et al. examined the efficacy of RU486 in the treatment of glucocorticoid dysregulation and fear-related abnormalities induced by traumatic stress; however, the long-term persistence of these effects remains unknown [21]. To better understand the possible advantages and disadvantages of pharmacological interventions for cognition, future studies should examine the long-term effects of these treatments. With regard to the intricate and diverse aspects of cognitive processes, articles comprising the Special Issue offer significant and instrumental perspectives. However, the underlying mechanisms and potential interventions for memory-related disorders remain largely unknown. Further investigation into the domains and knowledge gaps examined in these papers may contribute to the advancement of knowledge regarding memory and learning processes.

## 4. Conclusions

Preclinical research and computational medicine are important adjuncts to human studies to understand the neurobiological basis of cognitive functions and disorders [45,46,47,48,49,50,51,52,53,54]. Researchers can use these models to simulate cognitive mechanisms and investigate the complex interactions between genetics, environment, pharmacology, and comorbidities [55,56,57,58,59,60,61,62,63,64,65]. This Special Issue advances our understanding of the pathomechanisms underlying normal and pathological conditions, aids in the evaluation of potential treatments, and provides insights into the efficacy of therapies [66]. Preclinical models aid in translating laboratory findings to clinical cognitive impairment and shed light on their underlying abnormal functions according to translational research [67,68,69,70,71,72,73,74]. Furthermore, by allowing the use of tailored treatments for memory-related disorders, this approach will contribute to the advancement of personalized medicine [71,75,76,77,78,79,80,81]. It also enables the investigation of structural changes in the brain and advances imaging techniques for clinical use [82,83]. Preclinical research and computational medicine are critical for unraveling the complexities of neurological and mental disorders, providing critical insights, facilitating treatment testing, and paving the way for novel therapeutics and personalized medicine [84,85,86,87,88,89].

Neuropharmacological research is critical for this multidisciplinary endeavor. The investigation of how drugs and compounds interact with complex neural networks found in preclinical models allows for a better understanding of potential therapeutic agents [90,91,92,93]. These findings will help guide the future development of pharmacological interventions targeting specific molecular pathways implicated in neuropsychiatric disorders [94,95]. Researchers are investigating novel drug candidates, investigating their safety profiles, and evaluating their efficacy in alleviating the symptoms of conditions such as cognitive impairments associated with mental illnesses and comorbidities [96,97,98,99,100]. Advanced imaging techniques have greatly aided research on neuropsychiatric symptoms [101]. Neuroimaging research has linked these conditions to changes in the brain structure and function [90,102,103]. These imaging modalities have the potential to provide valuable insights into the pathophysiology of the disorders under investigation, and aid in the diagnosis of rare clinical cases. Furthermore, neuropharmacological approaches complement the broader scope of preclinical research, allowing for a more thorough investigation of the genetic, environmental, and pharmacological factors that influence mental health [55,57,104,105,106,107,108,109,110]. It allows for the faster identification of potential drug targets and the development of personalized medicine approaches tailored to individuals’ unique neurochemical profiles [111].

This Special Issue covers a wide range of topics related to memory and learning research and provides a comprehensive view of cutting-edge research in this field. Clinical implications and pharmacological interventions for memory disorders are discussed, along with the molecular and cellular mechanisms of synaptic plasticity and memory formation. Using a wide range of experimental approaches and analytical tools, the authors explored the neural correlates and molecular mechanisms of cognitive processes across a wide range of species, brain regions, and settings. The findings reported in these papers advance our understanding of the complex and dynamic nature of memory and learning while also opening up new avenues for future research and applications. We hope that this Special Issue will generate new dialogue and research on this fascinating and important topic among academics and wider society.

## Data Availability

Data sharing is not applicable to this article.

## Abbreviations

LTP	long-term potentiation
NMDA	N-methyl-D-aspartate
PFC	prefrontal cortex
